# Carbon nitride–TiO_2_ hybrid modified with hydrogenase for visible light driven hydrogen production†
†Electronic supplementary information (ESI) available. See DOI: 10.1039/c5sc02017d



**DOI:** 10.1039/c5sc02017d

**Published:** 2015-06-29

**Authors:** Christine A. Caputo, Lidong Wang, Radim Beranek, Erwin Reisner

**Affiliations:** a Christian Doppler Laboratory for Sustainable SynGas Chemistry , Department of Chemistry , Cambridge University , Lensfied Road , Cambridge CB2 1EW , UK . Email: reisner@ch.cam.ac.uk ; http://www-reisner.ch.cam.ac.uk; b Faculty of Chemistry and Biochemistry , Ruhr-Universität Bochum , Universitätsstraße 150 , 44780 Bochum , Germany

## Abstract

A system consisting of a [NiFeSe]–hydrogenase (H_2_ase) grafted on the surface of a TiO_2_ nanoparticle modified with polyheptazine carbon nitride polymer, melon (CN_*x*_) is reported. This semi-biological assembly shows a turnover number (TON) of more than 5.8 × 10^5^ mol H_2_ (mol H_2_ase)^–1^ after 72 h in a sacrificial electron donor solution at pH 6 during solar AM 1.5 G irradiation. An external quantum efficiency up to 4.8% for photon-to-hydrogen conversion was achieved under irradiation with monochromatic light. The CN_*x*_–TiO_2_–H_2_ase construct was also active under UV-free solar light irradiation (*λ* > 420 nm), where it showed a substantially higher activity than TiO_2_–H_2_ase and CN_*x*_–H_2_ase due, in part, to the formation of a CN_*x*_–TiO_2_ charge transfer complex and highly productive electron transfer to the H_2_ase. The CN_*x*_–TiO_2_–H_2_ase system sets a new benchmark for photocatalytic H_2_ production with a H_2_ase immobilised on a noble- and toxic-metal free light absorber in terms of visible light utilisation and stability.

## Abstract 2

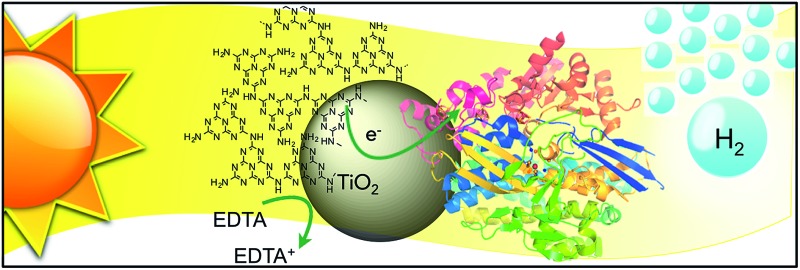
Solar light driven hydrogen production with a heterogenised hydrogenase on a carbon nitride–TiO_2_ hybrid is reported that sets a new benchmark for photo-H_2_ production.

## Introduction

The use of efficient electrocatalysts in artificial photocatalytic schemes has been an area of recent interest for the conversion of protons to hydrogen using sunlight. Specifically, the use of redox enzymes in photocatalytic schemes highlights the importance of investigating the compatibility of biological systems with light harvesting materials and testing the stability of the resultant bio-hybrid assemblies.^1^ Hydrogenases (H_2_ases) are the most efficient noble-metal free electrocatalysts for H_2_ production and achieve a turnover frequency (TOF) of more than 1000 s^–1^ with a small overpotential.^2^ H_2_ases also show impressive H_2_ production rates and yields in sacrificial photocatalytic schemes in pH neutral aqueous solution.^1*a*^ In these systems, a photoexcited light absorber provides electrons to the protein *via* an internal wire, the iron–sulfur electron relay, to the active site where proton reduction occurs. Examples are the immobilization of a H_2_ase on Ru-sensitised TiO_2_,^3^ on Cd-based quantum dots^4^ as well as homogeneous systems using the H_2_ase with a covalently linked photosystem I^5^ or in combination with an organic dye,^6^ and multi-component systems with a dye and a soluble redox mediator.^7^

Polymeric carbon nitride (polyheptazine or melon, herein CN_*x*_) is a promising visible-light absorber for the photocatalytic generation of H_2_.^8^ We have recently reported the use of CN_*x*_ as a light harvesting material in combination with a H_2_ase and a H_2_ase-inspired synthetic Ni catalyst for solar H_2_ generation.^9^ The CN_*x*_–H_2_ase system showed sustained catalysis with a turnover number (TON) of more than 50 000 after 70 h solar light irradiation. However, this hybrid system suffered from a weak interaction between the H_2_ase and the CN_*x*_ surface, and consequently, poor electron transfer from CN_*x*_ to the H_2_ase. Furthermore, CN_*x*_–H_2_ase only showed efficient H_2_ production up to wavelengths of approximately 420 nm and therefore only limited visible light harvesting capabilities.

**Fig. 1:**
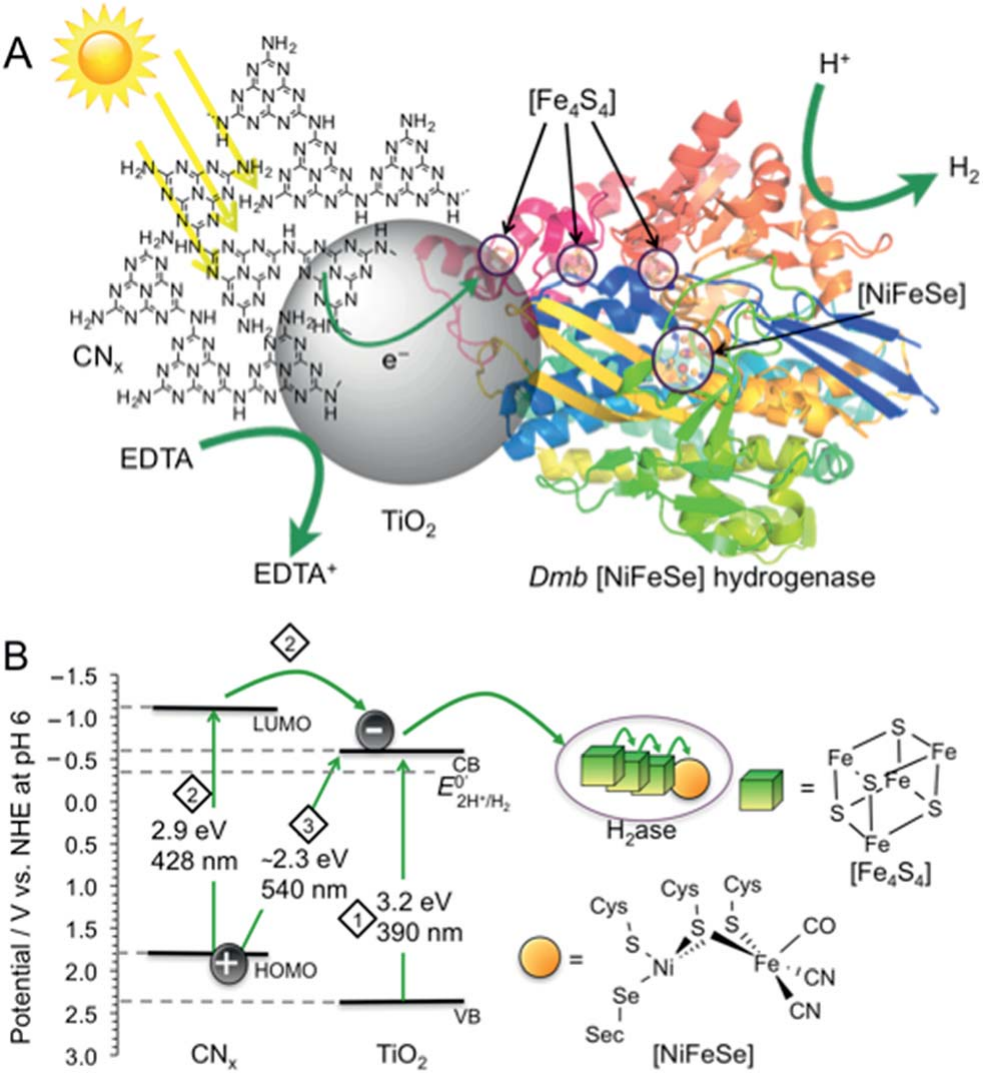
(A) Schematic representation of photo-H_2_ production with *Dmb* [NiFeSe]–H_2_ase (PDB ID : ; 1CC1)^14^ on CN_*x*_–TiO_2_ suspended in water containing EDTA as a hole scavenger. (B) Irradiation of CN_*x*_–TiO_2_ can result in photo-induced electron transfer by three distinct pathways: (1) TiO_2_ band gap excitation (2) excitation of CN_*x*_ (HOMO_CN_*x*__–LUMO_CN_*x*__), followed by electron transfer from LUMO_CN_*x*__ into the conduction band of TiO_2_ (CB_TiO_2__). (3) Charge transfer excitation with direct optical electron transfer from HOMO_CN_*x*__ to CB_TiO_2__. The CB_TiO_2__ electrons generated through pathways 1 to 3 are then transferred *via* the [Fe_4_S_4_] clusters to the [NiFeSe] H_2_ase active site.

Here, we selected a hybrid material consisting of TiO_2_ (Hombikat UV 100, anatase, BET surface area: 300 m^2^ g^–1^, crystallite size < 10 nm) surface-modified with CN_*x*_ polymer as a light absorbing hybrid material for the photocatalytic system with a H_2_ase for three main reasons (Fig. 1; see ESI and Fig. S1† for synthesis and characterisation). Firstly, CN_*x*_–TiO_2_ can be readily prepared on a gram scale by heating TiO_2_ nanoparticles in the presence of urea, an inexpensive and sustainable material.^10^

Secondly, CN_*x*_–TiO_2_ provides us with substantially improved solar light harvesting performance compared to individual CN_*x*_ and TiO_2_. *Band gap excitation* of TiO_2_ (pathway 1; Fig. 1) efficiently utilises the UV spectrum (band gap of 3.2 eV for anatase TiO_2_ with CB_TiO_2__ at approximately –0.6 V *vs.* NHE at pH 6).^11^ A significant portion of the visible spectrum is utilised with CN_*x*_–TiO_2_ as it can, upon photo-excitation of CN_*x*_, perform *photoinduced electron transfer* from the LUMO_CN_*x*__ to CB_TiO_2__ (pathway 2). In addition, *direct optical electron transfer* can occur from the HOMO_CN_*x*__ (with contributions of molecular orbitals formed upon interaction of CN_*x*_ with TiO_2_)^12^ directly to the CB_TiO_2__ (pathway 3), extending the absorption even further into the visible region (up to 540 nm). This absorption pathway 3 is based on strong coupling between CN_*x*_ covalently grafted onto TiO_2_, resulting in strong charge-transfer absorption. Conclusive evidence of this charge-transfer includes previously reported spectroscopic, photoelectrochemical, and theoretical investigations.^12,13^ The generated CB_TiO_2__ electrons provide the H_2_ase with an overpotential of approximately 0.2 V for proton reduction.

Thirdly, the H_2_ evolution catalyst employed in this study, *Desulfomicrobium baculatum* (*Dmb*) [NiFeSe]–hydrogenase is not only known for its high H_2_ evolution activity, lack of H_2_ inhibition and O_2_-tolerance,^6,14b,14c,15^ but also for its *titaniaphilicity*.^3*a*^ This high affinity of the enzyme to adsorb strongly to TiO_2_ stems presumably from a protein surface rich in glutamatic and aspartic acid residues close to the distal [Fe_4_S_4_] cluster, which act as anchor sites to TiO_2_ and allow for stable binding and efficient electron flow into the hydrogenase active site (Fig. 1A).^1a,3a^ Thus, the CN_*x*_–TiO_2_ hybrid is expected to support a more robust H_2_ase-particle interaction than with CN_*x*_ alone, which would result in improved charge transfer and ultimately increased catalytic turnover for H_2_ production.

## Results and Discussion

Photocatalytic systems were assembled by dispersing CN_*x*_–TiO_2_ particles in an aqueous electron donor solution (0.1 M; 2.98 mL) in a photoreactor vessel (headspace volume: 4.74 mL; see ESI† for experimental details). The vessel was sonicated under air (15 min) before sealing and purging with an inert gas (2% CH_4_ in N_2_). The H_2_ase (16.5 μL, 3 μM) was then added and the photo-reactor purged again to ensure anaerobic conditions. The stirred suspension was irradiated at 25 °C with a solar light simulator (air mass 1.5 global filter, *I* = 100 mW cm^–2^) and the headspace H_2_ was quantified at regular time intervals by gas chromatography against the internal CH_4_ standard. The conditions were optimised for maximum turnover frequency (TOF_H_2_ase_) by varying the electron donor and pH of the solution (Table S1; Fig. S2 and S3†). Optimised conditions consisted of ethylenediamine tetraacetic acid (EDTA; 0.1 M) as the electron donor at pH 6. A ratio of semiconductor (5 mg unless otherwise noted) to H_2_ase (50 pmol) was used for ease of comparison to previously reported photosystems with *Dmb* [NiFeSe]–H_2_ase.^3,6,9^

**Fig. 2:**
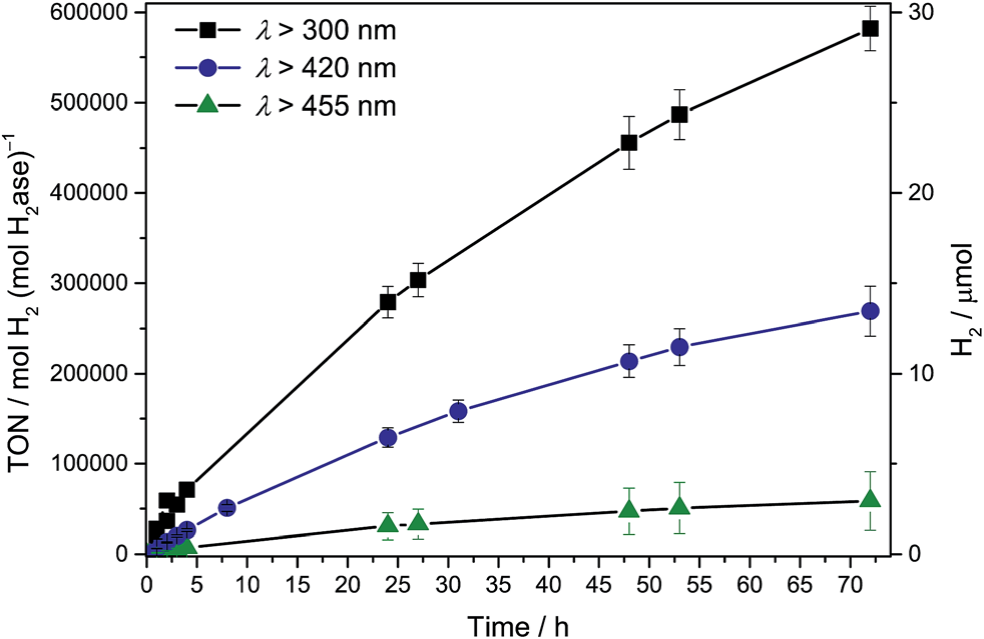
Photocatalytic H_2_ production with *Dmb* [NiFeSe]–H_2_ase (50 pmol) with CN_*x*_–TiO_2_ (5 mg) in EDTA (pH 6, 0.1 M, 3 mL) under AM 1.5G irradiation at an intensity of 1 Sun at *λ* > 300, 420 and 455 nm.

Solar (UV-visible) irradiation (*λ* > 300 nm) of CN_*x*_–TiO_2_–H_2_ase under standard conditions generated an initial TOF_H_2_ase_ of (2.8 ± 0.3) × 10^4^ h^–1^ or 8 s^–1^ with the production of 5.85 ± 0.59 μmol H_2_ after 4 h and 28 ± 3 μmol H_2_ with an overall TON_H_2_ase_ > (5.8 ± 0.6) × 10^5^ after 72 h (Fig. 2 and S4†). Negligible amounts of H_2_ were detected in the absence of H_2_ase, CN_*x*_–TiO_2_ or EDTA. UV band gap excitation of TiO_2_ did not result in the accumulation of O_2_, which suggests that holes generated upon UV band gap excitation of TiO_2_ are either efficiently quenched by EDTA directly or scavenged after being trapped by CN_*x*_.

To qualitatively determine the contributions from the three excitation pathways in Fig. 1B, irradiation was also performed with different long-pass filters. The CN_*x*_–TiO_2_–H_2_ase system was studied under visible light irradiation at *λ* > 420 nm to study the contribution of CN_*x*_ to light absorption (pathways 2 & 3) without the contribution of intrinsic absorption by TiO_2_ (pathway 1). A photoactivity with an initial TOF_H_2_ase_ of 6353 ± 635 h^–1^ was observed, which results in the generation of 1.31 ± 0.13 μmol H_2_ after 4 h. After 72 h, 13 ± 1 μmol of H_2_ were generated with a TON_H_2_ase_ of more than (2.6 ± 0.3) × 10^5^ (Fig. 2).

Subsequently, irradiation was carried out at *λ* > 455 nm to investigate the contribution of the direct charge-transfer from the HOMO_CN_*x*__ to CB_TiO_2__ to the photoactivity. A TOF_H_2_ase_ of 1096 ± 175 h^–1^ with the evolution of 0.26 ± 0.06 μmol H_2_ after 4 h and 2.9 ± 1.6 μmol H_2_ after 72 h was observed, which corresponds to 17% of the visible light activity. This suggests that all three pathways in Fig. 1B contribute to the UV-vis photoactivity, whereas pathways 2 and 3 are responsible for the visible-light response of CN_*x*_–TiO_2_–H_2_ase. Previous investigations of CN_*x*_–TiO_2_ hybrids have shown that their activity is limited by the strong electronic coupling between CN_*x*_ and TiO_2_ leading not only to intense visible light absorption but also to fast back electron transfer (primary recombination).^13,16^

In order to study the role of TiO_2_ as heterogeneous electron relay in CN_*x*_–TiO_2_–H_2_ase in more detail, a sample of CN_*x*_–ZrO_2_ (15 mg) was also tested with the H_2_ase. The negative CB_ZrO_2__ at approximately –1.35 V *vs.* NHE at pH 6, prevents electron injection from LUMO_CN_*x*__ (approximately –1.25 V *vs.* NHE at pH 6).^17^ This band level mismatch allowed us to demonstrate that spatial proximity of surface-bound H_2_ase to CN_*x*_ alone cannot promote productive electron transfer as no H_2_ was observed with CN_*x*_–ZrO_2_–H_2_ase (*λ* > 300 nm; Fig. S4†). Thus, charge transfer from the LUMO_CN_*x*__ into CB_ZrO_2__ (pathway 2) is not possible, nor is the direct electron transfer from HOMO_CN_*x*__ to CB_ZrO_2__ (pathway 3), which are crucial to the formation of H_2_ with the hybrid material.

For comparison, H_2_ production was also tested with CN_*x*_ (5 mg) and H_2_ase (50 pmol) in the absence of metal oxide under standard conditions. A TON_H_2_ase_ of 14852 ± 1485 was obtained after 4 h with an initial TOF of 6288 ± 649 h^–1^ when irradiated with UV-visible light (*λ* > 300 nm, Table S1†). Under visible light irradiation (*λ* > 420 nm), a TON_H_2_ase_ of 2375 ± 267 was observed after 4 h and no H_2_ was produced at *λ* > 455 nm, demonstrating the substantially enhanced activity with CN_*x*_–TiO_2_–H_2_ase compared to CN_*x*_–H_2_ase at all wavelengths (Fig. S4†).

Experiments were also performed with TiO_2_–H_2_ase. While the system showed comparable activity under UV-visible irradiation due to efficient band gap excitation of TiO_2_ (pathway 1), it showed significantly reduced activity under visible only irradiation at *λ* > 420 nm and displayed negligible H_2_ yields at *λ* > 455 nm compared to CN_*x*_–TiO_2_–H_2_ase (Fig. S4†).^9^ Thus, UV-band gap excitation of TiO_2_ dominates the absorption of the CN_*x*_–TiO_2_–H_2_ase hybrid material under UV-light irradiation, which becomes less significant under visible irradiation.

The effect of light intensity on the photocatalytic activity (*λ* > 300 nm) was studied by employing neutral density filters. A photoactivity of approximately 90% remained when employing a 50% absorbance filter (50 mW cm^–2^) and 44% of activity remained with an 80% filter (20 mW cm^–2^; Fig. S5†). The initial non-linear decrease in activity implies that the system is not limited by light at 1 Sun intensity as has been observed previously with synthetic H_2_ evolution catalyst-modified Ru dye-sensitised TiO_2_ systems.^18^

The CN_*x*_–TiO_2_–H_2_ase system sets a new benchmark for visible light driven and prolonged H_2_ production with a heterogenised H_2_ase without the need for expensive or toxic materials.^3,4,9^ A part of this improvement can be attributed to the direct optical electron transfer (pathway 3) within CN_*x*_–TiO_2_, which draws the absorption of solar light significantly into the visible spectrum.

**Fig. 3:**
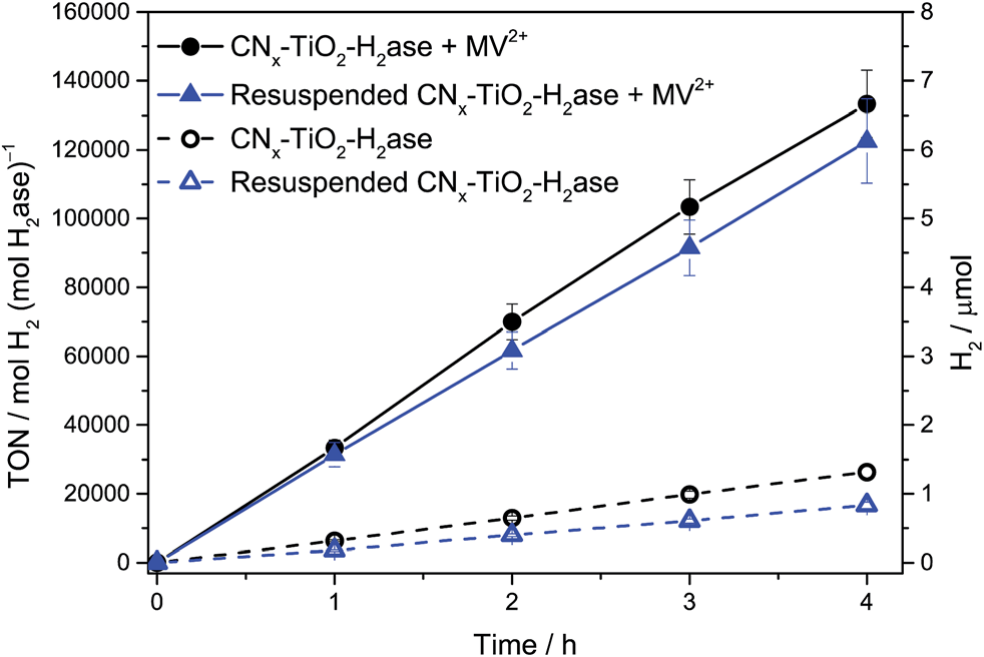
Photocatalytic H_2_ production using *Dmb* [NiFeSe]–H_2_ase (50 pmol) in EDTA (pH 6, 0.1 M, 3 mL) with CN_*x*_–TiO_2_ (5 mg) under optimised conditions before and after centrifugation and re-suspension in fresh EDTA buffer solution followed by 1 Sun irradiation (*λ* > 420 nm). Results are also shown in the presence and absence of redox mediator, methyl viologen (MV^2+^).

The enzyme loading onto CN_*x*_–TiO_2_ was calculated based on the BET surface area of 111 m^2^ g^–1^, a crystallite surface area of ∼314 nm^2^ per particle and an estimation that approximately one-quarter of the surface area of TiO_2_ is accessible for the enzyme to adsorb. This equates to ∼0.1 H_2_ase per particle of CN_*x*_–TiO_2_. The approximate 1 : 10 enzyme : particle ratio allows the H_2_ase to function at the maximum rate (*i.e.*, TOF) as the maximum electron flux of conduction band electrons is directed towards a single enzyme. To qualitatively determine the amounts of surface-bound and solubilised H_2_ase in the optimised system, H_2_ase (50 pmol) was loaded onto CN_*x*_–TiO_2_ (5 mg) in aqueous EDTA solution by stirring under N_2_ for 15 min. The suspension was centrifuged and the supernatant decanted (see ESI† for experimental details). The CN_*x*_–TiO_2_–H_2_ase pellet was re-dispersed in fresh EDTA solution (3 mL, 0.1 M, pH 6) and the photocatalytic vessel purged with 2% CH_4_ in N_2_. The suspension was then irradiated (*λ* > 420 nm) and H_2_ production monitored (Fig. 3). The H_2_ production activity was nearly identical to a sample that was not centrifuged, both in the presence and absence of methyl viologen (MV^2+^, see below), indicating that attachment of H_2_ase to CN_*x*_–TiO_2_ is essentially quantitative. The substantially improved adsorption of the enzyme on the TiO_2_ surface compared to the inert CN_*x*_ polymer therefore also contributes to the increased activity of CN_*x*_–TiO_2_–H_2_ase compared to CN_*x*_–H_2_ase. Previously an 88% decrease in photoactivity was observed with the poorly interacting CN_*x*_–H_2_ase after centrifugation and re-dispersion in fresh electron donor buffer.^9^

The external quantum efficiency (EQE) of the CN_*x*_–TiO_2_–H_2_ase system was measured by applying narrow band pass filters (*λ* = 360 ± 10 nm; *I* = 2.49 mW cm^–2^ and 400 ± 10 nm; *I* = 4.34 mW cm^–2^; see ESI† for experimental details). UV-irradiation gave an EQE of approximately 4.8% and under visible irradiation an EQE of 0.51% was obtained. These values are more than a 10-fold improvement over the UV and visible EQE for the CN_*x*_–H_2_ase system,^9^ which can be attributed to the improved light absorption (Fig. S6†) and increased electron transfer rate due to adsorption of the H_2_ase onto the particle surface.

We previously showed that a significantly increased photoactivity was observed under standard conditions using CN_*x*_–H_2_ase upon addition of an excess of the redox mediator MV^2+^, producing up to 77 μmol H_2_ after 69 h of UV-visible irradiation.^9^ A long-term experiment with H_2_ase (50 pmol), CN_*x*_–TiO_2_ (5 mg) and added MV^2+^ (5 μmol) in aqueous EDTA (0.1 M) at pH 6 was performed with both *λ* > 300 nm light and with visible light only (*λ* > 420 nm). Under UV-visible irradiation after 72 h, the CN_*x*_–TiO_2_–MV–H_2_ase system produced 193 μmol H_2_ with a TON_H_2_ase_ of > 3.8 × 10^6^ and an initial TOF_H_2_ase_ of 35 s^–1^ (Fig. S7†). Under visible-light only, 66 μmol H_2_ was produced with a TON_H_2_ase_ of 1.3 × 10^6^ and an initial TOF_H_2_ase_ of 9 s^–1^ (Fig. S8†). The ratio of the amount of hydrogen produced in the presence and absence of MV^2+^ can be used to estimate the relative efficiency of the charge transfer from material to H_2_ase. Under full spectrum irradiation (*λ* > 300 nm) with CN_*x*_–H_2_ase the ratio was found to be 22, whereas for both TiO_2_–H_2_ase and CN_*x*_–TiO_2_–H_2_ase systems the ratio was 5. This strongly supports the fact that there is a significant improvement in the charge transfer from a TiO_2_-based material to H_2_ase. In addition, this ratio remains constant when the wavelength of light used is restricted to the visible region (*λ* > 420 nm).

The H_2_ production rates in the presence of MV^2+^ are significantly higher than those obtained in the absence of MV^2+^. The blue colour of the vials containing MV^2+^ is indicative of the formation of reduced MV^+^˙ in solution (Fig. S9†). By comparison, addition of MV^2+^ to the previously reported Ru-dye-sensitised TiO_2_–H_2_ase system caused a slight decrease in activity, which was attributed to the decreased availability of electrons for the H_2_ase and the absorption of incident photons by MV^+^˙.^3*a*^ Here, solubilised MV^+^˙ does not limit light absorption by CN_*x*_–TiO_2_ significantly and is able to efficiently donate electrons to surface-bound H_2_ase, resulting in increased H_2_ production. This result implies that interfacial electron transfer from CN_*x*_–TiO_2_ to H_2_ase is still not fully optimised in this system, where the orientation of the H_2_ase is not fully ‘directed’. Ideally, the distance from the CN_*x*_–TiO_2_ surface to the [Fe_4_S_4_] electron transport chain should be minimised and an improved orientation of the enzyme would allow trapping of CB_TiO_2__ electrons more efficiently for maximised turnover.^19^

Favourable electron transfer kinetics at the CN_*x*_–TiO_2_–H_2_ase interface can be assumed based on previous reports. Electron transfer in the order of 10^7^ s^–1^ was reported from CdS nanorods to an [FeFe]–H_2_ase isolated from *Clostridium acetobutylicum*.^4*c*^ In addition, a long lived photo-excited state lifetime of *τ*_1/2_ ∼ 0.8 s was previously reported for TiO_2_ conduction band electrons in a photocatalytic system with Ru dye-sensitised TiO_2_ and electron transfer to co-immobilised molecular cobaloxime catalysts occurred with *τ*_1/2_ ∼ 5 to 50 μs.^20^ Based on these reports, we can assume that a reasonably long-lived TiO_2_ conduction band electron is generated and that H_2_ase is capable of readily collecting these electrons.

## Conclusions

In summary, solar light driven H_2_ production with a semi-biological system consisting of TiO_2_ modified with polymeric CN_*x*_ and immobilised H_2_ase has been demonstrated. We have shown that by improving the surface interaction of the enzyme with the light harvesting CN_*x*_ material, specifically by adsorption of the enzyme onto the TiO_2_ surface, H_2_ generation is drastically improved. Another important factor is the improved visible light absorption by direct CN_*x*_ excitation (pathway 2) and CN_*x*_–TiO_2_ charge transfer (pathway 3), which enables high photoactivity. The CN_*x*_–TiO_2_–H_2_ase assembly achieved a TOF of 8 s^–1^ and TON of > 5.8 × 10^5^ after 72 h in the absence of an external soluble redox mediator, thereby setting a new benchmark for photochemical architectures based on abundant and non-toxic materials and a heterogenised H_2_ase. The additional use of the redox mediator MV^2+^ allowed for the photo-generation of H_2_ with a TOF of 35 s^–1^ and a TON of > 3.8 × 10^6^. This work advances the use of hybrid photocatalytic schemes by integrating highly active electrocatalysts with advanced light absorbing materials such as CN_*x*_–TiO_2_, which is shown to be compatible with H_2_ases in aqueous solution.
