# Supplementary material for: Carbon nitride–TiO2 hybrid modified with hydrogenase for visible light driven hydrogen production
Source: Chem Sci. 2015 Jun 29;6(10):5690–4. doi: 10.1039/c5sc02017d (PMC5512016; doi:10.1039/c5sc02017d)
Supplement: Supplementary file 1 [file SC-006-C5SC02017D-s001.pdf]

Electronic Supplementary Material (ESI) for Chemical Science.
This journal is © The Royal Society of Chemistry 2015

Electronic Supplementary Information (ESI)

Carbon Nitride-TiO_2 Hybrid Modified with Hydrogenase for Visible Light Driven Hydrogen Production

*Christine A. Caputo,[a] Lidong Wang,[b] Radim Beranek,[b] and Erwin Reisner[a]**

[a]Christian Doppler Laboratory for Sustainable SynGas Chemistry, Department of Chemistry, University of Cambridge, Lensfield Road, Cambridge CB2 1EW, UK

[b]Faculty of Chemistry and Biochemistry, Ruhr-Universität Bochum , Universitätstr. 150, 44780 Bochum, Germany

To whom correspondence should be addressed: email: reisner@ch.cam.ac.uk

Table of Contents:

	PAGE
Experimental Section	S2
References	S5
Tables S1 to S2	S6
Figures S1 to S9	S7

Experimental Section

Materials. Chemicals were purchased from commercial suppliers and used without further purification. Reagents for the analytical part of the work were of the highest available purity. The [NiFeSe]-hydrogenase from *Desulfomicrobium baculatum* (*Dmb* [NiFeSe]-H_2ase) was purified by a previously published method.[1] The preparation has a specific activity of 2115 µmol H_2 min^{-1} mg^{-1},[2] and the stock enzyme solution was diluted with an aqueous TEOA solution (0.1 M, pH 7) before photocatalytic experiments in an anaerobic glovebox. Polyheptazine (melon, CN_x) was prepared by heating melamine at 500 °C for 2 hours.[3] Note that the CN_x employed in this study displayed the same activity than a previously used CN_x from a different source.[4] CN_x-TiO_2 powders were synthesised as previously described.[3b] In short, 0.5 g of TiO_2 (Hombikat UV 100, Sachtleben Chemie, Germany, anatase, specific surface area (BET) 300 m^2 g^{-1}, crystallite size < 10 nm) was placed into a Schlenk tube connected via an adapter to a round-bottomed flask containing urea (1 g), and heated in a muffle oven for 30 min at 425 °C. Powders were washed and centrifuged with water three times and then dried at 60 °C overnight, giving samples denoted as CN_x-TiO_2. The specific surface area (BET) of CN_x-TiO_2 and of CN_x was 111 m^2 g^{-1} and 8 m^2 g^{-1}, respectively. CN_x-ZrO_2 was prepared using an analogous procedure with a urea heat treatment at 600 °C starting with ZrO_2 powders. ZrO_2 was prepared by basic hydrolysis (pH 9) of $ZrOCl_2$ (1 M) solutions, followed by filtration, drying, and calcination at 500 °C for 2 hours.

Photocatalysis Experiments. A standard photocatalytic experimental set-up was used as follows: CN_x-TiO_2 (5 mg) was added to a borosilicate glass tube containing a magnetic stir bar (total volume 7.74 mL). An aqueous electron donor solution (usually 0.1 M, 2.98 mL) was then added and the vessel sealed with a rubber septum. The suspension was sonicated for 20 min under air, followed by purging the suspension for 15 min with 2% CH_4 in N_2. *Dmb* [NiFeSe]-H_2ase was then added

(16.5 μL; 3 mM) and then the vial was purged with 2% CH_4 in N_2 for an additional 5 min. The vials were then placed in a water-jacketed glass rack and irradiated with 1 sun using a Xe lamp (Newport Oriel Solar Light Simulator, 1000 W, 100 mW cm^{-2}) with an air mass 1.5 global (AM 1.5G) filter in the absence or presence of a 420 nm or 455 nm UV-broad band cut-off filters (UQG Optics), with stirring. Headspace gases were sampled using Hamilton air-tight syringes by injecting 20 μL into the gas chromatograph (Agilent 7890A Series gas chromatograph equipped with a 5 Å molecular sieve column, N_2 carrier gas 3 mL $(min)^{-1}$ and an oven temperature of 45 °C and a thermal conductivity detector) at regular intervals. H_2 produced was quantified by comparison to the CH_4 internal standard.

Centrifugation Experiments. Photocatalytic experiments were set up by pre-loading the H_2ase (50 pmol) onto CN_x-TiO_2 (5 mg) by stirring the suspension under N_2 for 15 min in an aqueous EDTA solution (0.1 M, 1 mL). The suspension was then transferred to centrifuge tubes and spun down for 10 min (10,000 rpm). The supernatant was decanted (in air) and the pellet re-suspended in an aqueous EDTA solution (0.1 M, pH 6) and vortexed for 2 min. The suspension was returned to the borosilicate glass vials, sealed and purged with 2% CH_4 in N_2 for 10 min. The vessels were irradiated under 1 sun for 4 h and H_2 production monitored by gas chromatography.

Calculation of Hydrogenase Loading. The enzyme loading onto CN_x-TiO_2 was calculated based on the BET surface area of 111 m^2 g^{-1}, a crystallite size of $r = 5 \times 10^{-9}$ m and a conservative estimate that approximately one-quarter of the surface area of CN_x-TiO_2 is exposed TiO_2 that is accessible for the enzyme to adsorb. *SA* is surface area, *A* is Avogadro's number, *n* is the number of moles of hydrogenase used, *m* the number of particles of CN_x-TiO_2 used.

$$\text{Number of } \mathrm{CN_xTiO_2} \text{ particles}, m = \frac{(\text{BET of } \mathrm{CN_xTiO_2})(\text{g } \mathrm{CN_xTiO_2})(\% \, SA \text{ exposed } \mathrm{TiO_2})}{4\pi r^2}$$

$$= \frac{(111 \text{ m}^2\text{g}^{-1})(0.005 \text{ g})(0.25)}{4\pi(5\times10^{-9})^2 \text{ m}^2}$$

$$= 4.4 \times 10^{14} \text{ particles}$$

$$\therefore \text{Hydrogenase Loading} = \frac{nA}{m \; \mathrm{CN_xTiO_2} \text{ particles}}$$

$$= \frac{(50 \times 10^{-12} \text{ mol } \mathrm{H_2}\text{ase})(6.02 \times 10^{23} \; \mathrm{H_2}\text{ase mol}^{-1})}{4.4 \times 10^{14} \text{ particles}}$$

$$= 0.1 \; \mathrm{H_2}\text{ase enzymes per particle of } \mathrm{CN_xTiO_2}$$

Treatment of Data. All analytical measurements were performed at least in triplicate. A minimum standard deviation (σ) of 10% was assumed for all experiments even where triplicate runs gave a σ of less than 10%. The solar light source and the gas chromatograph were calibrated regularly to ensure reproducibility.

Calculation of External Quantum Efficiency (EQE). Hydrogen generation in a quartz cuvette containing excess H_2ase (50 pmol) with CN_x-TiO_2 (1.7 mg) suspended in EDTA (1 mL, 0.1 M, pH 6) was driven by blue light (λ = 400 ± 10 nm) of intensity I = 4.34 mW cm^{-2} and UV light (λ = 360 ± nm) of intensity I = 2.49 mW cm^{-2} at 25 °C using narrow bandpass filters (THOR Labs). The EQE can be calculated with the following formula:

$$\text{EQE (\%)} = (2 \cdot n_{H2} \cdot N_A \cdot h \cdot c)/(t_{irr} \cdot \lambda \cdot I \cdot A) \cdot 100$$

Where n_{H2} is the moles of H_2 photo-generated, N_A is the Avogardo constant, h is the Planck constant, c is the speed of light, t_{irr} is the irradiation time, and A is the irradiated area of the photoreactor (1 cm^2).

References

1 A. Volbeda, P. Amara, M. Iannello, A. L. De Lacey, C. Cavazza and J. C. Fontecilla-Camps, *Chem. Commun.*, 2013, **49**, 7061-7063.

2 E. C. Hatchikian, M. Bruschi and J. Le Gall, *Biochem. Biophys. Res. Commun.*, 1978, **82**, 451-461.

3 (a) B. V. Lotsch, M. Döblinger, J. Sehnert, L. Seyfarth, J. Senker, O. Oeckler and W. Schnick, *Chem. Eur. J.*, 2007, **13**, 4969-4980; (b) M. Bledowski, L. Wang, A. Ramakrishnan, O. V. Khavryuchenko, V. D. Khavryuchenko, P. C. Ricci, J. Strunk, T. Cremer, C. Kolbeck and R. Beranek, *Phys. Chem. Chem. Phys.*, 2011, **13**, 21511-21519.

4 C. A. Caputo, M. A. Gross, V. W. Lau, C. Cavazza, B. V. Lotsch and E. Reisner, *Angew. Chem. Int. Ed.*, 2014, **53**, 11538-11542.

Table S1. Solar light driven H_2 production with *Dmb* [NiFeSe]-H_2ase (50 pmol) on CN_x-TiO_2 (5 mg). Experiments were performed using CN_x-TiO_2 or other light harvesting particles (5 mg) in aqueous ascorbic acid (AA; 0.1 M), triethanolamine (TEOA; 0.1 M), EDTA (0.1 M) or citric acid (CA; 0.1 M) solution with H_2ase as a catalyst. Entry 6 represents optimised standard conditions under AM 1.5 G solar light irradiation (λ > 300 nm).

Entry	H_2ase / pmol; light absorber	pH	Electron donor	TON (4 h) ± σ / mol H_2 H_2ase^{-1}	H_2 (4 h) ± σ / µmol	Activity (1 h) / µmol H_2 (g CN_x-TiO_2)$^{-1}$ h^{-1}	TOF (1 h) ± σ / h^{-1}
**Different Electron Donors (UV-vis irradiation)**							
**1**	50; CN_x-TiO_2	6	AA	762 ± 72	0.04 ± 0.01	5 ± 1	516 ± 52
**2**	50; CN_x-TiO_2	10.5	TEOA	2378 ± 234	0.12 ± 0.01	9 ± 1	959 ± 96
**3**	50; CN_x-TiO_2	7	EDTA	52960 ± 5296	2.65 ± 0.27	132 ± 13	13217 ± 1322
**4**	50; CN_x-TiO_2	6	CA	7032 ± 1380	0.35 ± 0.07	21 ± 3	2097 ± 397
**Variation of pH values (UV-vis irradiation)**							
**5**	50; CN_x-TiO_2	5	EDTA	111491 ± 14138	5.57 ± 0.59	262 ± 39	26273 ± 3989
**6**	50; CN_x-TiO_2	6	EDTA	116953 ± 11695	5.85 ± 0.59	279 ± 28	27928 ± 2793
**7**	50; CN_x-TiO_2	7	EDTA	52960 ± 5296	2.65 ± 0.27	132 ± 13	13217 ± 1322
**Visible Light Irradiation (*l* > 420 nm)**							
**8**	50; CN_x-TiO_2	6	EDTA	26277 ± 2628	1.31 ± 0.13	63 ± 6	6353 ± 635
**9**	50; TiO_2	6	EDTA	12327 ± 1699	0.62 ± 0.08	21 ± 2	2093 ± 222
**10**	50; CN_x	6	EDTA	2317 ± 304	0.11 ± 0.01	8 ± 1	845 ± 98
**Visible Light Irradiation (*l* > 455 nm)**							
**10**	50; CN_x-TiO_2	6	EDTA	5152 ± 1119	0.26 ± 0.06	11 ± 1	1096 ± 175
**11**	50; TiO_2	6	EDTA	993 ± 193	0.05 ± 0.01	—	—
**12**	50; CN_x	6	EDTA	—#	—	—	—
**Control experiments (UV-vis irradiation)**							
**13**	0; CN_x-TiO_2	6	EDTA	—	0.35 ± 0.06	10 ± 2	—
**14**	50; CN_x-TiO_2	6	H_2O†	25587 ± 2558	1.28 ± 0.13	88 ± 9	8814 ± 881
**15**	50; no CN_x-TiO_2	6	EDTA	—	—	—	—
**16**	50; TiO_2	6	EDTA	156308 ± 15630	7.82 ± 0.76	364 ± 54	36418 ± 5126
**17***	50; CN_x-ZrO_2	6	EDTA	3450 ± 345	0.17 ± 0.02	4 ± 1	1054 ± 229
**18**	50; CN_x	6	EDTA	14852 ± 1485	0.74 ± 0.07	63 ± 7	6288 ± 649

#No H_2 detected by GC measurements (limit of detection < 0.01%).

†A small amount of sacrificial electron donor TEOA was present in this experiment (16.5 µL of 0.1 M TEOA H_2ase buffer solution).

*These experiments were carried out with 15 mg of CN_x-ZrO_2

Table S2. Solar light driven (λ > 300 nm) H_2 production using CN_x-TiO_2 (5 mg) in an aqueous EDTA solution (0.1 M, pH 6) with *Dmb* [NiFeSe]-H_2ase (50 pmol) under standard conditions after 1 h of irradiation in the presence of different neutral density filters.

Entry	TON (4 h) ± σ / mol H_2 H_2ase^{-1}	H_2 (4 h) ± σ / μmol	Activity / μmol H_2 (g CN_x-TiO_2)$^{-1}$ h^{-1}	TOF ± σ / h^{-1}
No additional neutral density filter was employed				
**1**	105305 ± 10531	5.3 ± 0.5	281 ± 28	28115 ± 2812
A neutral density filter (Absorbance 50%) was employed				
**2**	96462 ± 9646	4.8 ± 0.5	228 ± 23	22883 ± 2289
A neutral density filter (Absorbance 80%) was employed				
**3**	46091 ± 6145	2.3 ± 0.3	95 ± 12	9462 ± 1164

*Vials were placed 10 cm closer to the light source thus may have been irradiated with a higher intensity of light than that used in standard experiments, thus a control with no filter was also measured. These measurements were performed in triplicate.

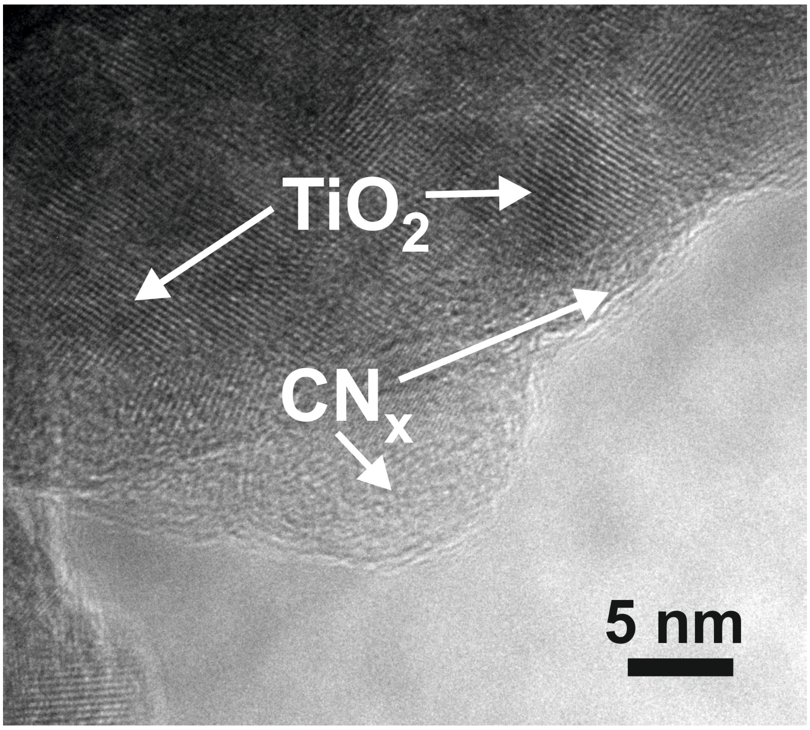

Figure S1. TEM of CN_x-TiO_2 material clearly showing CN_x on the TiO_2 surface. [M. Bledowski, L. Wang, S. Neubert, D. Mitoraj, R. Beranek, *J. Phys. Chem. C.* **2014**, *118*, 18951-18961; M. Bledowski, L. Wang, S. Neubert, A. Ramakrishnan, R. Beranek, *J. Mater. Res.* **2013**, *28*, 411.]

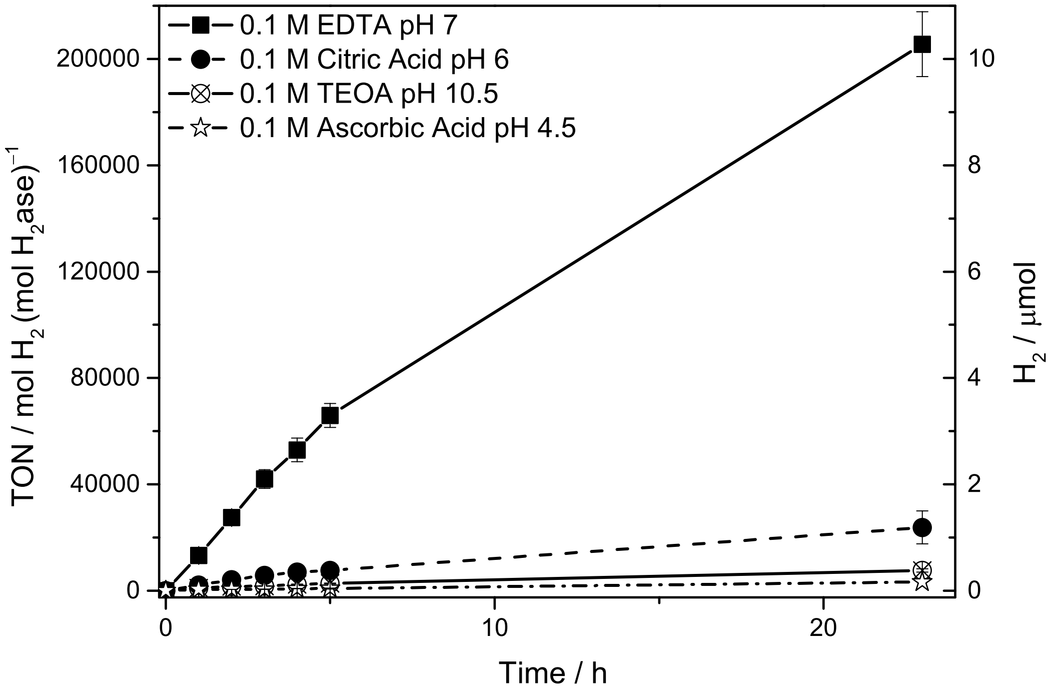

Figure S2. Photo-H_2 production with *Dmb* [NiFeSe]-H_2ase (50 pmol) with CN_x-TiO_2 (5 mg) in aqueous solutions with different sacrificial electron donors (0.1 M) during solar light irradiation (1 sun, 100 mW cm^{-2}, λ > 300 nm) at 25 °C.

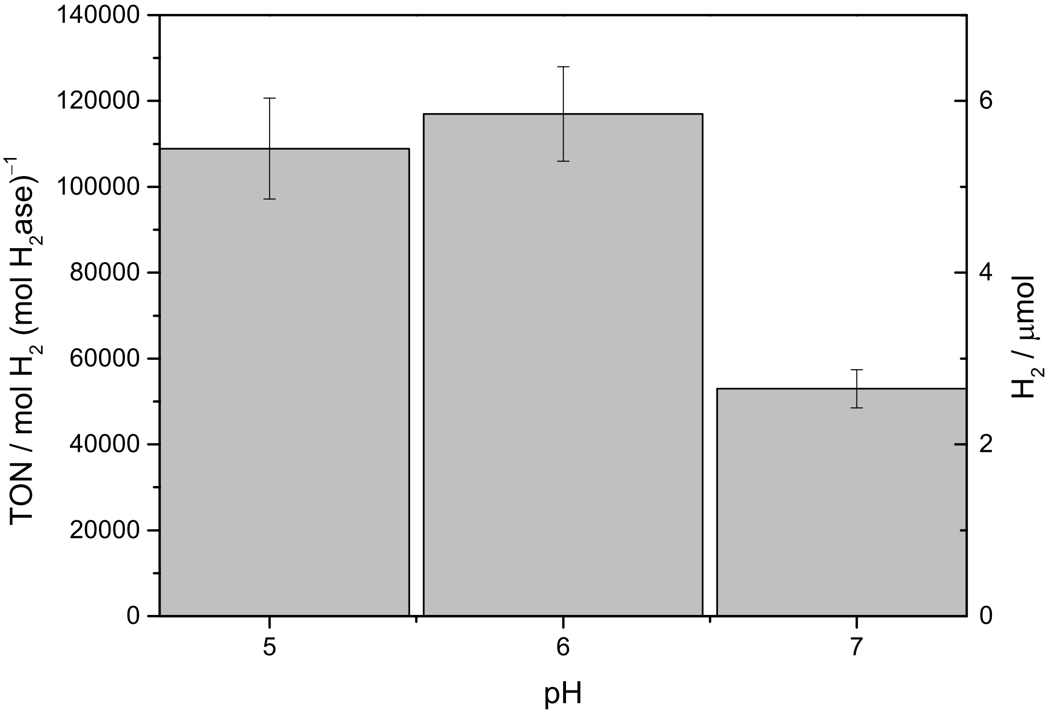

Figure S3. TON_{H2ase} for photo-H_2 production with *Dmb* [NiFeSe]-H_2ase (50 pmol) in an aqueous EDTA solution (0.1 M) with CN_x-TiO_2 (5 mg) at different pH values obtained after 4 h of solar light irradiation (1 sun, 100 mW cm^{-2}, λ > 300 nm) at 25 °C.

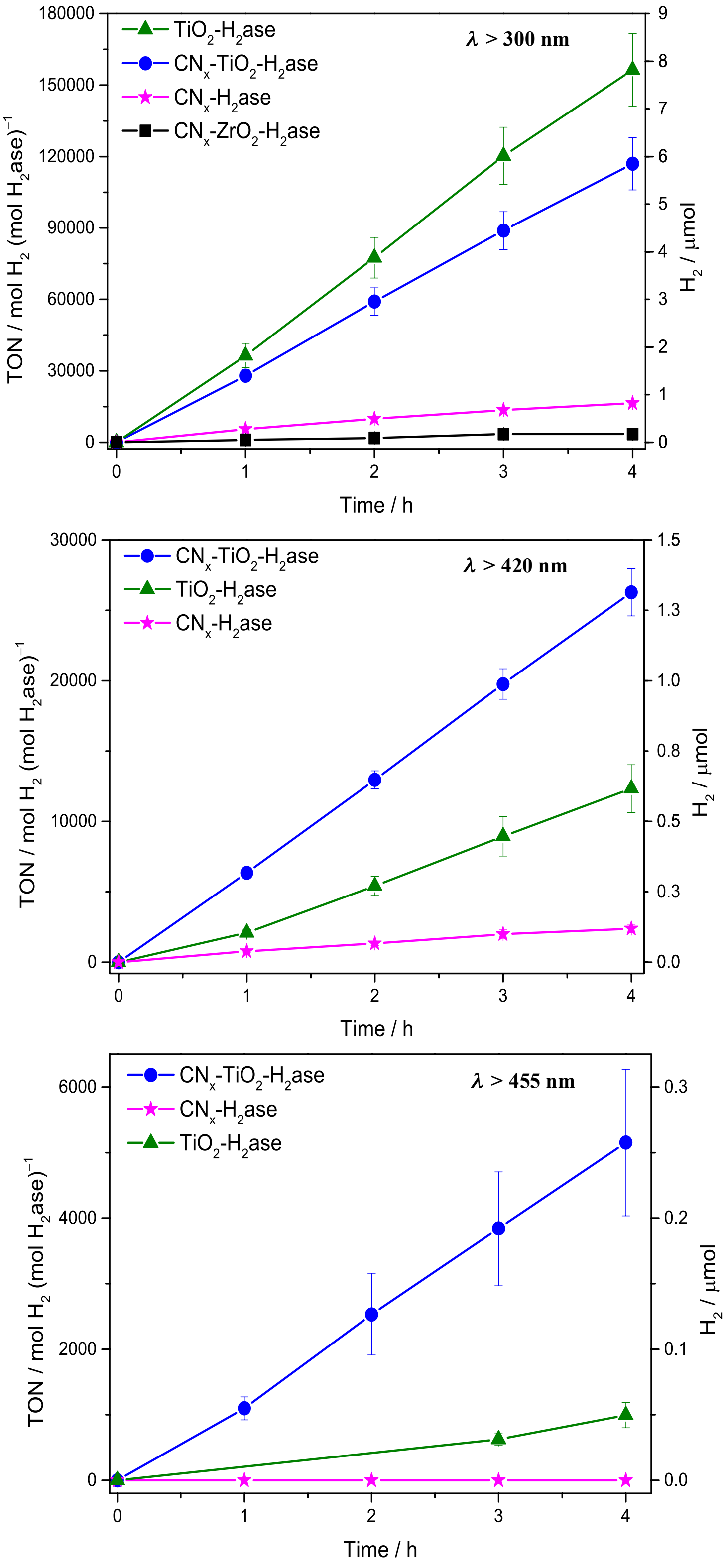

Figure S4. H_2 production under optimised conditions using *Dmb* [NiFeSe]-H_2ase (50 pmol) in EDTA (pH 6, 0.1 M, 3 mL) and CN_x-TiO_2, TiO_2, CN_x, (5 mg) or CN_x-ZrO_2 (15 mg) under 1 sun irradiation with $\lambda > 300$ nm, $\lambda > 420$ nm and $\lambda > 455$ nm light. Note that the amount of H_2 was below the detection limit after the first 2 h at $\lambda > 455$ nm with TiO_2-H_2ase.

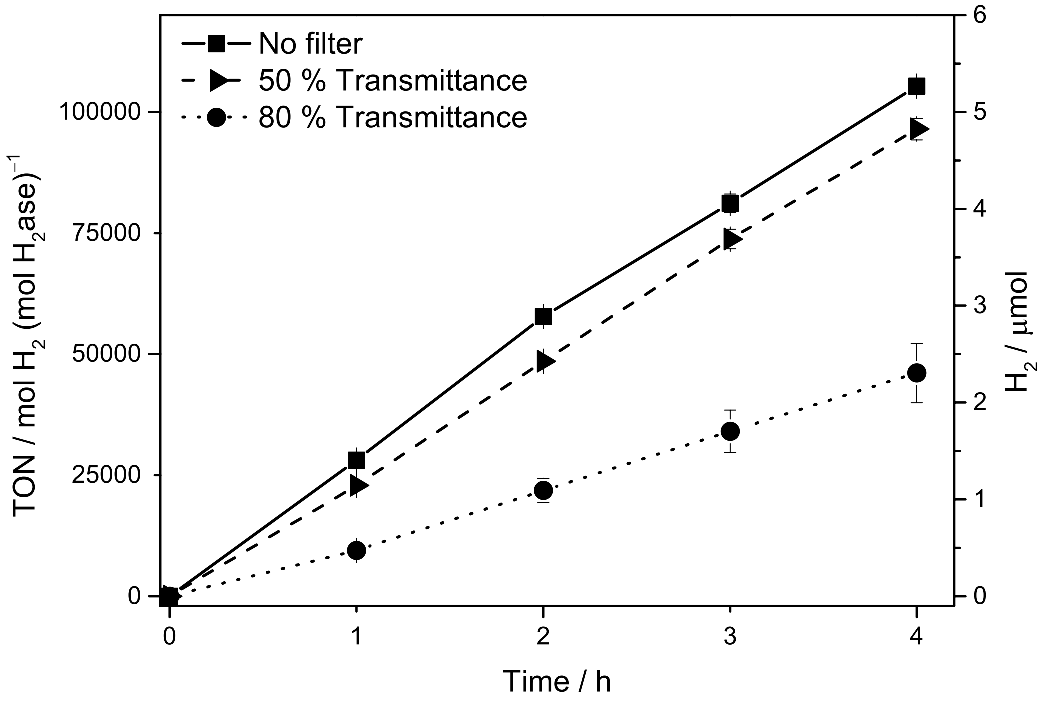

Figure S5. Photo-H_2 production using CN_x-TiO_2 (5 mg) in aqueous EDTA solution (0.1 M, pH 6) with *Dmb* [NiFeSe]-H_2ase (50 pmol) under 1 sun irradiation (100 mW cm^{-2}, λ > 300 nm, 25 °C) with the addition of different neutral density filters absorbing 50% and 80% of the incident light.

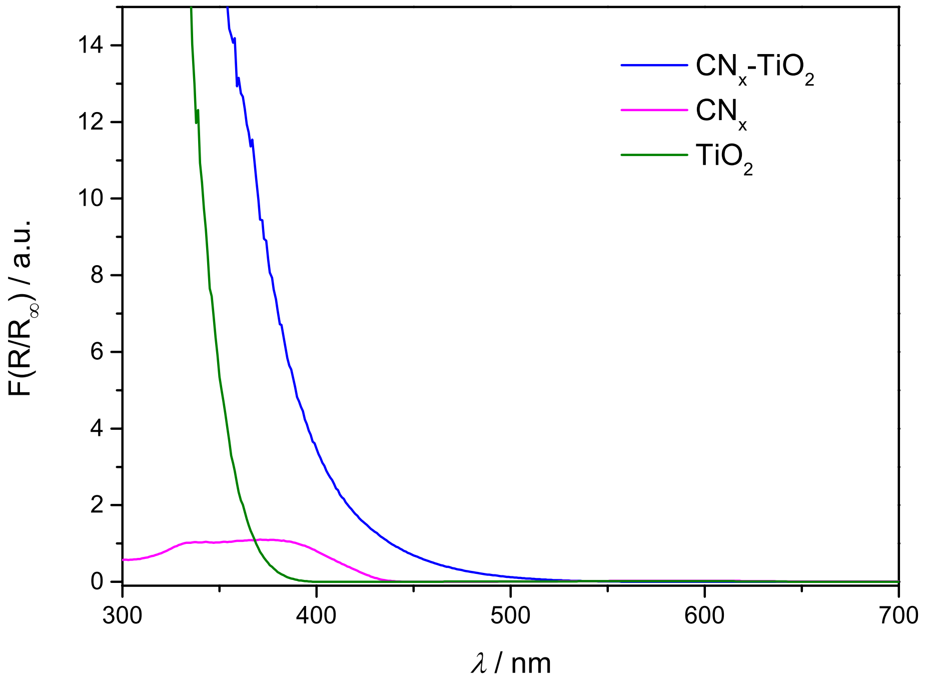

Figure S6. Diffuse reflectance UV-vis spectrum of CN_x-TiO_2, CN_x and TiO_2.

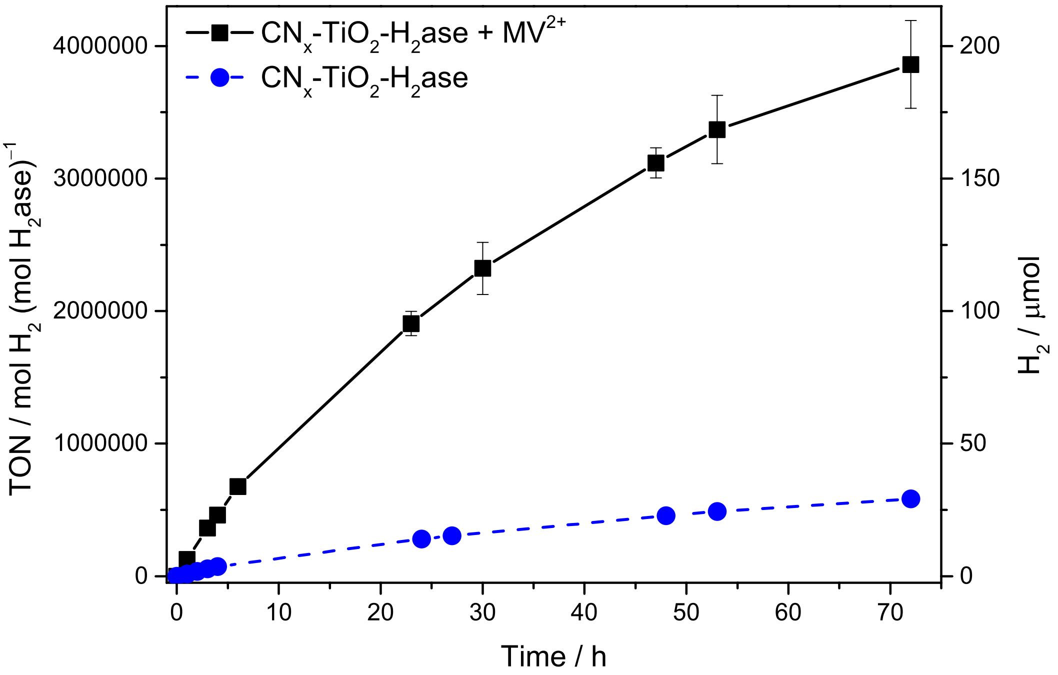

Figure S7. Long-term photo-H_2 production with *Dmb* [NiFeSe]-H_2ase (50 pmol) and CN_x-TiO_2 (5 mg) in aqueous EDTA solution (0.1 M, pH 6) in the presence and absence of methyl viologen (MV^{2+}; 5 µmol) under solar light irradiation (1 sun, 100 mW cm^{-2}, $\lambda > 300$ nm) at 25 °C.

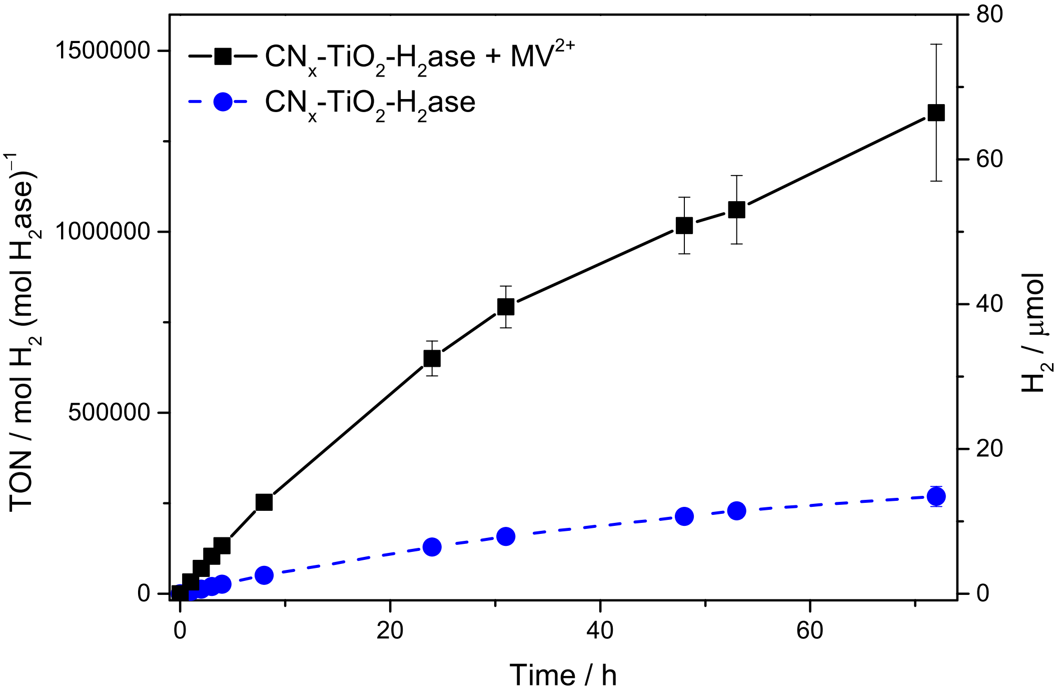

Figure S8. Long-term photo-H_2 production with *Dmb* [NiFeSe]-H_2ase (50 pmol) and CN_x-TiO_2 (5 mg) in aqueous EDTA solution (0.1 M, pH 6) in the presence and absence of methyl viologen (MV^{2+}; 5 µmol) under visible light irradiation (1 sun, 100 mW cm^{-2}, $\lambda > 420$ nm) at 25 °C.

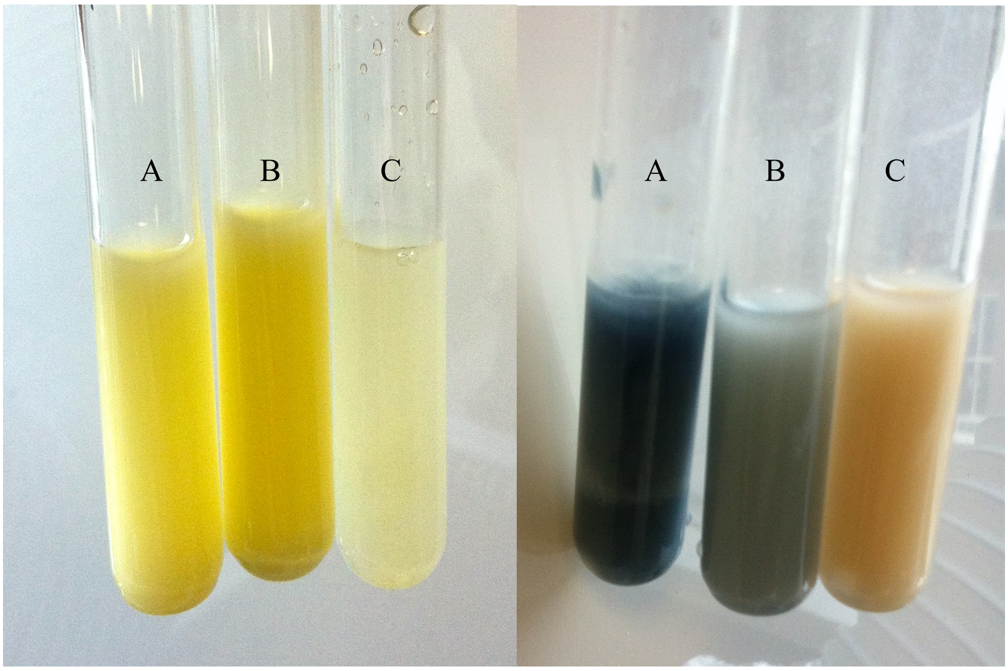

Figure S9. Colour of suspensions of (A) CN_x-TiO_2-H_2ase + MV^{2+}, (B) CN_x-TiO_2-H_2ase, (C) CN_x-H_2ase before (left) and after 72 hours of solar light (λ > 300 nm) exposure (right).

End of ESI
